# Antibodies against 4 Atypical Post-Translational Protein Modifications in Patients with Rheumatoid Arthritis

**DOI:** 10.3390/diagnostics12020352

**Published:** 2022-01-29

**Authors:** Lorena Rodríguez-Martínez, Cristina Regueiro, Sámer Amhaz-Escanlar, Carmen Pena, Paloma Herbello-Hermelo, Antonio Moreda-Piñeiro, Javier Rodriguez-Garcia, Antonio Mera-Varela, Eva Pérez-Pampín, Antonio González

**Affiliations:** 1 Experimental and Observational Rheumatology and Rheumatology Unit, Instituto de Investigacion Sanitaria-Hospital Clínico Universitario de Santiago (IDIS), 15706 Santiago de Compostela, Spain; lorenamarinoalvarez@gmail.com (L.R.-M.); cristina.regueiro.exposito@rai.usc.es (C.R.); egodoylopez@hotmail.com (C.P.); antonio.mera.varela@sergas.es (A.M.-V.); evappampin@gmail.com (E.P.-P.); 2 Department of Orthopedic Surgery and Traumatology, Instituto de Investigación Sanitaria-Hospital Clínico Universitario de Santiago (IDIS), 15706 Santiago de Compostela, Spain; sameramhaz@gmail.com; 3 Trace Element, Spectroscopy and Speciation Group (GETEE), Strategic Grouping in Materials (AEMAT), Department of Analytical Chemistry, Nutrition and Bromatology, Faculty of Chemistry, Universidade de Santiago de Compostela, 15782 Santiago de Compostela, Spain; paloma.herbello@usc.es (P.H.-H.); antonio.moreda@usc.es (A.M.-P.); 4 Department of Clinical Analysis, Instituto de Investigación Sanitaria-Hospital Clínico Universitario de Santiago (IDIS), 15706 Santiago de Compostela, Spain; javier.rodriguez.garcia@sergas.es; 5 Department of Medicine, Faculty of Medicine, Universidade de Santiago de Compostela, 15705 Santiago de Compostela, Spain

**Keywords:** rheumatoid arthritis, autoantibodies, post-translational protein modifications, chlorination, non-enzymatic glycation, nitration, oxidation, carbonylation, homocysteinylation, citrullination

## Abstract

Patients with rheumatoid arthritis (RA) show autoantibodies against post-translational protein modifications (PTMs), such as anti-citrullinated protein antibodies. However, the range of recognized PTMs is unknown. Here, we addressed four PTMs: chlorination, non-enzymatic glycation, nitration, and homocysteinylation, identified as targets of atypical RA autoantibodies in studies whose protocols we have followed. The modified antigens included collagen type II, an extract of synovial proteins and a selection of peptides. We interpreted the results according to the optical density (OD) obtained in an enzyme-linked immunosorbent assay ( ELISA) with the modified antigen and the corrected OD obtained after subtracting the reactivity against the unmodified antigen. The results showed evidence of specific antibodies against glycated collagen type II, as the corrected ODs were higher in the 182 patients with RA than in the 164 healthy controls (*p* = 0.0003). However, the relevance of these antibodies was doubtful because the magnitude of the specific signal was small (median OD = 0.072 vs. 0.027, respectively). There were no specific antibodies against any of the other three PTMs. Therefore, our results showed that the four PTMs are not inducing a significant autoantibody response in patients with RA. These results indicated that the repertoire of PTM autoantigens in RA is restricted.

## 1. Introduction

Rheumatoid arthritis (RA) is a chronic autoimmune disease of complex etiology and pathogenesis [1]. It is characterized by inflammation at multiple joints leading to pain, morning stiffness, joint swelling, and functional disability. Further, RA has a systemic component of inflammation and extra-articular manifestations that may involve the skin, vessels, and lungs or increase the risk of cardiovascular disease, infections, and malignancies. The systemic component is also revealed by the presence of autoantibodies in the blood and synovial fluid of the patients that, in many cases, precedes the clinical onset of RA [1,2,3].

The autoantigens recognized by the RA antibodies are multiple and still incompletely defined [1,2,3]. All of the known autoantigens have a wide distribution in organs and tissues, and many of them are proteins with post-translational protein modifications (PTMs). The best studied examples are the citrullinated, carbamylated, and acetylated proteins [3,4]. In addition, well established autoantigens include the malondialdehyde and malondialdehyde-acetaldehyde adduct containing proteins, although the antibodies against these are less specific for RA [4,5]. All of these PTMs affect many proteins and take place in many tissues in a variety of physiological and pathological situations. However, it is suspected that PTMs occurring in synovial tissue and favored by inflammation or oxidative processes could be more relevant for RA. The identity of the proteins that are modified is less important for the binding of the autoantibodies than the PTM itself, meaning that the patient can sera bind multiple different peptides with the same PTM [4,6,7,8].

We still do not know if the range of PTMs recognized as autoantigens in RA is wide, or restricted to a few PTMs [3]. Some studies suggest a large range of PTMs by showing a large prevalence of antibodies against new PTM [9,10,11,12,13,14]. The antibodies against them can be considered atypical because they are not yet well established as RA autoantibodies. In fact, the most well replicated of those analyzed here has only been reported in three non-independent studies [11,13,14]. The independent validation of atypical anti-PTM antibodies, therefore, will confirm additional targets and mechanisms of RA autoimmunity, and contribute to defining their role as biomarkers. These expectations are supported by previous experience with the anti-citrullinated protein antibodies, which have been critical for advances in RA pathogenesis and are the main laboratory biomarkers in RA [1,2,3]. Other well established autoantibodies against PTMs also show potential value as biomarkers. For example, the presence of anti-carbamylated protein antibodies or anti-acetylated peptide antibodies can be used to improve RA disease classification [15,16].

In this study, we aimed to explore four atypical anti-PTM antibodies that have been described in RA as recognizing the following PTM: protein chlorination by hypochlorous acid (anti-HOCl) [11,13], which is one of the reactive oxygen species found in arthritic joints; protein non-enzymatic glycation and formation of advanced glycation end products (AGEs) by ribose (anti-NEG) [11,13,14]; the tyrosine nitration of peptides (anti-3-NT) or, more broadly, the nitration of proteins (anti-NO_2_) by peroxynitrite [10,11], which is a reactive nitrogen species increased in inflamed joints; and the homocysteinylation (anti-Hcy) by a metabolite of homocysteine, homocysteine-thiolactone [9,12]. We treat the four separately, although the three first PTMs are oxidations, because the reactions used to produce them result in complex modifications showing only partial overlap [17,18,19,20,21,22,23]. The four PTMs are over-represented in arthritic joints and RA patient plasma reflecting the increased oxidative stress, formation of AGEs or homocysteinylation processes [9,18,22,24,25]. Therefore, confirmation of the specificity of RA autoantibodies against them will imply their participation in autoimmunity, tissue damage, and pathology. In addition, the anti-HOCl-CII antibodies have been described as extremely sensitive and specific for early RA [13], a feature that could be of great interest if confirmed.

## 2. Materials and Methods

### 2.1. Patients and Samples

We analyzed serum samples from 325 patients with RA meeting the 1987 ACR or the 2010 ACR/EULAR classification criteria [26,27], and 197 healthy controls (median age = 69 years, IQR = 63–75; 60% women) enrolled at the Hospital Clínico Universitario de Santiago. We considered the 182 RA patients with samples taken less than two years after symptom onset as an early RA subset (ERA), whereas the remaining 143 RA patients formed an established RA subset. The clinical characteristics of the two RA subsets are presented in Table 1. The controls were selected for good general health and the absence of known musculoskeletal, inflammatory, or autoimmune diseases. Smoking status was defined as never been a smoker or ever smoked (past or current). We also used synovial tissue collected during knee joint replacement surgery from three patients with osteoarthritis. The study was approved by the Autonomous Research Ethics Committee of Galicia (Ref. 2014/387 and 2017/514). All of the participants provided written informed consent. In addition, this work was conducted according to the relevant guidelines and regulations (Declaration of Helsinki, the Belmont Report, the Spanish Laws of Biomedical Research 14/2007, and Data Protection 3/2018).

### 2.2. In Vitro Production of the Four PTMs

We have produced the four PTMs using in vitro reactions, as previously described [10,11,28] and summarized in Table 2. A corresponding procedure without the modifying agent was conducted for each of the four PTMs to produce the unmodified protein controls.

The collagen type II (CII) was obtained from bovine cartilage as a highly purified lyophilized powder (#804001, MD Bioproducts, Oakdale, MN, USA). This protein, at 2 mg/mL in PBS 1X pH 7.4, was used to obtain oxidized CII (HOCl-CII) and glycated CII (NEG-CII) [11]. The first of these modified proteins was produced with 1 mM freshly prepared hypochlorous acid (HOCl) at 37 °C for 24 h in sterile conditions protected from the light. In turn, the NEG-CII was produced with 2 M ribose at 37 °C for 24 h in sterile conditions protected from the light. Subsequently, the two modified proteins were dialyzed in PBS 1X pH 7.4 for 24 h at 4 °C with at least 3 changes of buffer.

We produced the other two PTMs on a protein extract obtained from synovial tissue (SynP). The extract was obtained by homogenizing the tissue samples using a Polytron PT 1200 E (Kinematica AG) on 3 mL/mg tissue of lysis buffer (sodium orthovanadate 1 mM, Triton X-100 1X, Tris-HCl 50 mM, NaCl 250 mM, sodium pyrophosphate 30 mM, EDTA 5 mM, NaF 100 mM, DTT 1 mM, Protease Inhibitor Cocktail 1X [PIC, P8340, Merck, Germany]). The homogenate was incubated for 30 min on ice and then filtered with Amicon^®^ ultrafiltration 3 kDa filters (Millipore, Merck, Germany) at 10,000× *g* at 4 °C. The supernatant containing the synovial proteins (SynP) was collected and stored at −80 °C. For the production of the nitrated proteins (NO_2_-SynP), we used 4 mg/mL SynP in PBS 1X pH 3.5 that reacted with 10 mM sodium peroxynitrite (NaONO_2_, Sigma, Merck, Germany) at 37 °C for 24 h, as previously described [10]. The modified proteins were concentrated by ultrafiltration with the 3 kDa Amicon^®^ filters and a washing step with PBS 1X pH 9 to remove unreacted NaONO_2_ and reaction by-products. The Nε-homocysteinylated SynP (Hcy-SynP) were produced by the reaction of the SynP at 10 mg/mL with 1 mM L-homocysteine thiolactone hydrochloride (L-Hcy-thiolactone, Alfa Aesar, Thermo Scientific, Waltham, MA, USA) in 0.05 M potassium phosphate buffer pH 7.4, 0.2 mM EDTA [28]. The reaction was carried out at 37 °C for 16 h. The resulting Hcy-SynP were concentrated through ultrafiltration on 3 kDa Amicon^®^ filters, as described above, to remove the unreacted L-Hcy-thiolactone.

Additionally, we modified albumin, either bovine (BSA) or human (HSA), or fetal bovine serum (FBS) for the set-up of the four post-translational modifications and assessment of the reactions performance [10,11,28]. In addition, Hcy-HSA was used as the antigen in a preliminary assay of the Hcy PTM. The protein concentrations were determined using Bradford’s reagent (Bio-Rad, Hercules, CA, USA). Following these reactions, the four PTMs were characterized as detailed in Appendix A. All of the proteins were stored in single-use aliquots at −20 °C until their use as antigens, except CII that was stored at 4 °C protected from light.

### 2.3. Selection of Ten Nitrated Peptides

We complemented the exploration of the anti-NO_2_ antibodies using 10 nitrated peptides taken from relevant proteins in RA with a molecular weight of approximately 80 kDa corresponding to the main nitrated synovial proteins in patients with RA [1,2,3,4,6,7,8,25]. Within the resulting list, we identified 10 peptides with tyrosine residues likely to be nitrated in vivo. These peptides were concordantly selected by two software algorithms: iNitro-Tyr predictor with a Δ-value > 0.4 [29], and GPS-YNO2 1.0 with a score > 0.9 at the medium threshold [30]. The tyrosine to be nitrated was placed in the middle position of a 21 mer amino acid sequence (Table 3). This design was chosen because it performs well for ELISA antigens [31]. The 10 peptides were synthesized by Schafer-N (Copenhagen, Denmark) in a native and a nitrated version, all of them biotinylated at the N-terminus. The synthesized peptides were subjected to quality control by Schafer-N that included high-performance liquid chromatography (HPLC) followed by mass spectrometry (MS). Two nitrated peptides that showed less than 70% purity were discarded.

### 2.4. Determination of Autoantibodies

The RF-IgM (cut-off 20 units/mL) was determined by nephelometry (IMMAGE Immunochemistry System, Beckman Coulter). The anti-CCP antibodies were determined using the EDIA anti-CCP test kit (Euro-Diagnostica, Malmö, Sweden) with a cut-off of 5 units/mL and according to the manufacturer’s instructions. The remaining autoantibodies were measured using a homemade ELISA. The assays were performed according to the specifications of the studies where they had been reported [10,11,12,13]. Therefore, we describe here the general protocol for all the ELISA, and we signal the details for each autoantibody in the next paragraph. The assays began by coating the 96-well ELISA Nunc MarxiSorp plates (Thermo Scientific, Waltham, MA, USA) with the antigens (the modified and control proteins in separate plates) at 5 μg/mL in PBS pH 7.4 (100 μL) overnight at 4 °C. The washes (3×) were performed using 200 μL PBS-Tween 0.05% (PBS-T). The blocking reagent was 2% non-fat dry milk in PBS that was incubated for 2 h at RT. The sera (100 μL at 1:100) were incubated for 2 h at 37 °C. The goat anti-human IgG Fcγ-specific antibody conjugated with alkaline phosphatase (AP) (Jackson ImmunoResearch, West Grove, PA, USA) was incubated at 1:2000 for 2 h at 37 °C and revealed using the p-nitrophenyl phosphate substrate (PNPP) (Gerbu Biotechnik GmbH, Wieblingen, Germany) in diethanolamine buffer (Thermo Scientific, Waltham, MA, USA). The optical density (OD) was read at 405 nm after 30 min using a Multiskan EX Microplate Reader (Thermo Scientific, Waltham, MA, USA). All of the samples were assayed in duplicate using the same batch of modified and control antigens to avoid bias due to antigen preparation. In addition, the sera from the patients and healthy subjects were run on each plate to exclude inter-plate bias. Each ELISA plate contained positive and negative control sera for assessing assay variability. Raw OD and corrected OD values were used in the analyses. The corrected OD values were equal to the OD against the modified antigen minus the OD against the native antigen. The cut-off for positivity was set at the 98th percentile of the OD found in healthy controls.

The anti-HOCl-CII and anti-NEG-CII ELISA were assayed following the general protocol. For the anti-NO_2_-SynP antibodies, the antigens were coated at 2.5 μg/mL (50 μL) in 0.1 M carbonate buffer pH 9.6; the blocking reagent was 1% BSA + PBS-T; the sera (50 μL at 1:100) were incubated for 2 h at RT; and the secondary antibody was the AP-conjugated goat anti-human IgG F(ab’)2 (Jackson ImmunoResearch, West Grove, PA, USA) antibody (50 μL at 1:10,000) and it was incubated for 1 h at RT before adding the substrate. Regarding the antibodies against the nitrated peptides, the ELISA was done using pools of the 8 peptides that passed quality control at a concentration of 4 mg/mL each. The two pools (3-NT and control) were coated on streptavidin-coated high binding capacity 96-well plates (Pierce, Thermo Fisher Scientific, Waltham, MA, USA) at 2.5 μg/mL of each peptide. Other aspects of the ELISA were as described for the NO_2_-SynP except that the secondary antibody was an AP-conjugated goat anti-human IgG (H + L) (Jackson ImmunoResearch, West Grove, PA, USA) antibody diluted 1:5000 that was incubated for 2 h at RT. Finally, the specific characteristics of the anti-Hcy-SynP ELISA were as follows: the coating was prepared at 10 μg/mL (100 µL/well) in 0.1 M carbonate buffer pH 9.6; the blocking reagent was 0.5% fish gelatin in PBS and was applied for 6 h at 4 °C; the sera (100 μL at 1:200) were incubated overnight at 4 °C; and the secondary antibody was the AP-conjugated goat anti-human IgG F(ab’)2 (Jackson ImmunoResearch, West Grove, PA, USA) antibody (100 μL at 1:20,000), which was incubated for 3.5 h at 4 °C.

### 2.5. Statistical Analysis

Descriptive statistics of the continuous variables are shown as the mean ± standard deviation (SD) or the median with the interquartile range, as indicated in each case. For nominal variables, the frequency with the corresponding percentage is provided. The continuous variables were compared with the Mann–Whitney U test, whereas the Chi-squared test for 2 × 2 contingency tables was used for the dichotomous ones. *p*-values below 0.05 were considered statistically significant. The data were analyzed using Statistica software (StatSoft, Tulsa, OK, USA), and the figures were made using GraphPad Prism v5 (La Jolla, CA, USA).

## 3. Results

We verified that the reactions had successfully produced the four PTMs (Appendix A). The checks included the demonstration of comparable proteins to the previously reported HOCl-CII and NEG-CII [11]; and proteins showing the predicted characteristics for NO_2_-SynP and Hcy-SynP. After passing the verification, the modified proteins were used as antigens in the assays.

### 3.1. Prevalence of the Anti-HOCl-CII and Anti-NEG-CII Antibodies in the Patients with Early RA

**Figure 1:**
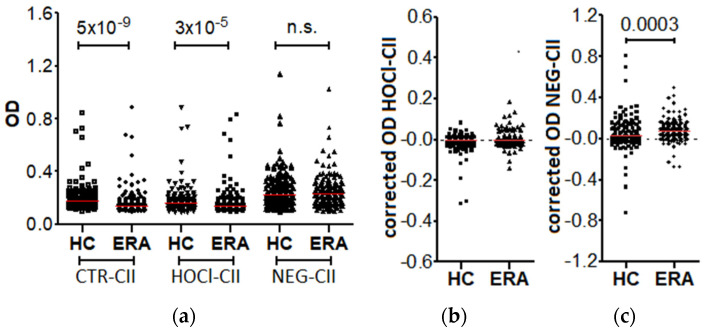
Reactivity against modified CII in patients with early RA (ERA) and healthy controls (HC). (**a**) Reactivity measured as OD before correction; (**b**) anti-HOCl-CII; and (**c**) anti-NEG-CII specific reactivities, obtained after subtracting the OD against the CTR-CII. The red line represents the median OD value in each group. n.s. = non significant.

The presence of antibodies was evaluated as an uncorrected reactivity against the modified CII and as a corrected OD after subtracting the immunoreactivity against unmodified CII (anti-CTR-CII) (Figure 1, Supplementary Table S1).

The results showed a higher level of reactivity against the CTR-CII in the healthy controls than in the RA patients (median OD = 0.174 vs. 0.143, *p* = 5.0 × 10^−9^). The increase was small but significant. A similar difference was observed with the uncorrected OD of anti-HOCl-CII antibodies: a significantly greater level was seen in the healthy controls than in the ERA patients (median OD = 0.160 vs. 0.141, *p* = 3.1 × 10^−5^). The corrected OD showed no anti-HOCl-CII reactivity larger than zero in either the ERA patients or the healthy controls (median OD = −0.004 and −0.002, respectively). Therefore, there was no specific reactivity against the HOCl-CII and no difference between the patients and controls. In contrast, the uncorrected OD of the anti-NEG-CII antibodies were not different between the ERA patients and the controls (median OD = 0.234 vs. 0.221, *p* = 0.5), whereas the corrected OD were significantly larger in the ERA patients than in the controls (median OD = 0.072 vs. 0.027, *p* = 0.0003). However, this increased immunoreactivity should be taken with caution due to the small magnitude of the specific signal.

### 3.2. Antibodies against NO_2_-SynP and 3-NT Peptides in RA Patients

**Figure 2:**
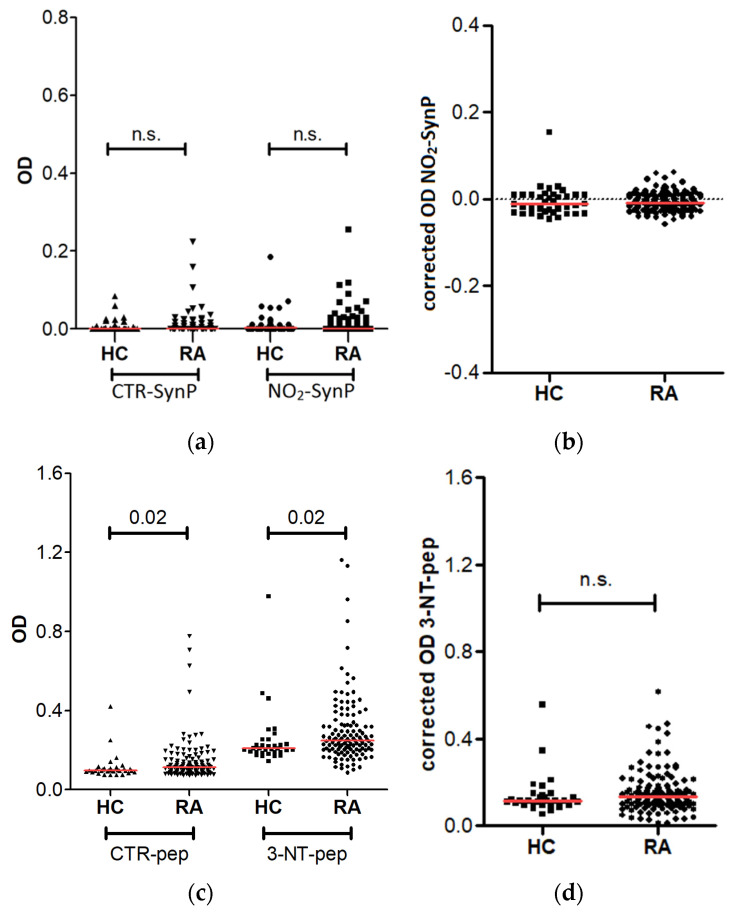
Reactivity against nitrated proteins and peptides in RA patients and healthy controls. (**a**) Uncorrected; and (**b**) corrected OD of the reactivity against NO_2_-SynP; (**c**) uncorrected; and (**d**) corrected OD of the reactivity against a 3-NT-pep. The red line represents the median OD value in each group. n.s. = non significant.

The anti-NO_2_-SynP and anti-3-NT-pep antibodies were evaluated in the sera of 143 patients with established RA and 56 healthy controls. Initially, we analyzed the immunoreactivity against the NO_2_-SynP, but the results showed little signal in all of the tested conditions (Figure 2a,b, Supplementary Table S1). That is, the levels were barely over zero already in the uncorrected OD of the anti-NO_2_-SynP antibodies (median OD = 0.018 and 0.011 in the RA patients and the controls, respectively). In addition, the corrected OD of the anti-NO_2_-SynP was negative after subtracting the OD obtained against the CTR-SynP (median OD = −0.009 and −0.011 in the RA patients and the controls, respectively).

Because of these results, we completed the study by exploring the reactivity against eight synthetic nitrated peptides (3-NT-pep). These peptides were selected from relevant proteins in RA and for their likelihood of in vivo nitration as described in the Material and Methods section and in Table 3; namely: enolase, fibrinogen, vimentin, 14-3-3 η, and histones H3 and H4. The ELISA coated with the peptides showed larger OD in the RA patients than in the healthy controls against the unmodified peptides (Figure 2c,d). The increase was small but significant (median OR = 0.111, vs. 0.097, *p* = 0.02). The same difference was observed in the uncorrected OD against 3-NT-pep. That is, the OD were larger in the RA patients than in the controls (median OD = 0.250 vs. 0.209, *p* = 0.02). This increase was accounted for by the immunoreactivity against the unmodified peptides, as shown in the corrected OD. In effect, the corrected OD anti-3-NT-pep were not different between the RA patients and the healthy controls (median OD = 0.137 vs. 0.113, *p* = 0.1). Therefore, we did not detect specific reactivity against nitration PTM, either on proteins or on peptides, in the serum of RA patients.

### 3.3. Prevalence of Anti-Hcy-SynP Antibodies in Patients with RA

**Figure 3:**
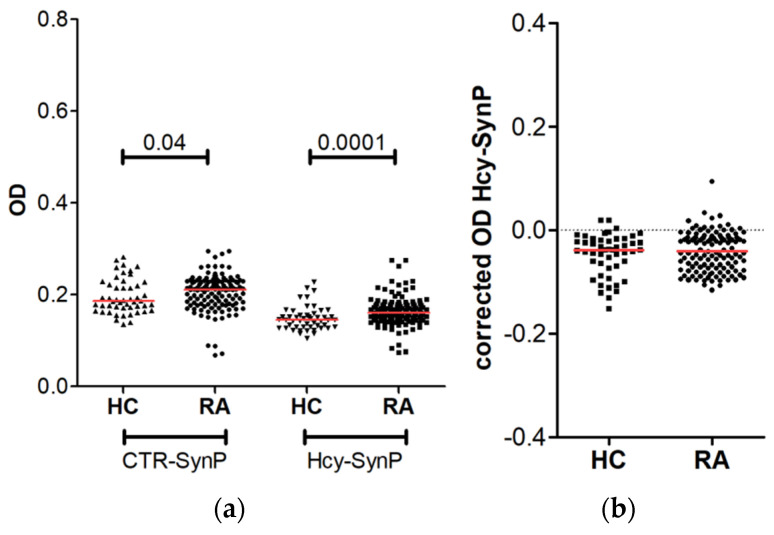
Reactivity against homocysteinylated SynP in RA patients and healthy controls. (**a**) Reactivity measured as OD before subtracting the OD against CTR-SynP; (**b**) specific reactivity against Hcy-SynP after correction. The red line represents the median OD value in each group.

We also analyzed the antibodies against homocysteinylated proteins. In a preliminary assay with a subset of the samples, we used Hcy-HSA as the antigen following a previous report [12], but the low reactivity obtained (Supplementary Table S2) motivated us to use the SynP extract as the antigen. The definitive ELISA was done using the sera of the 143 patients with established RA and of the 56 healthy controls. We assessed the immunoreactivity against the Hcy-SynP, homocysteinylated proteins, and the CTR-SynP, unmodified proteins (Figure 3, Supplementary Table S1). The OD against the CTR-SynP were larger in the RA patients than in the healthy controls (median OD = 0.211 vs. 0.186, *p* = 0.04). This result does not match that shown in the analysis of the anti-NO_2_ reactivity because the CTR-SynP were processed separately for each PTM: in parallel with homocysteinylation and nitration, respectively; and assayed with the corresponding ELISA protocols. In turn, the levels of the anti-Hcy-SynP antibodies before correction were also higher in the RA patients than in the controls (median OD = 0.160 vs. 0.145, *p* = 0.0001) (Figure 3 and Supplementary Table S1). However, the OD were lower than those obtained against the CTR-SynP, a feature that resulted in a negative corrected OD both in the RA patients and in the controls (median OD = −0.041 and −0.038, respectively). Therefore, our results showed no specific immunoreactivity against protein homocysteinylation.

## 4. Discussion

Our main results are the absence of specific antibodies against chlorinated-CII (HOCl-CII), nitrated synovial proteins (NO_2_-SynP), nitrated peptides (3-NT-pep), and homocysteinylated synovial proteins (Hcy-SynP) and the lack of a meaningful level of anti-ribose glycated CII (NEG-CII) antibodies. These results question the significance of the reactivity against the four PTMs in patients with RA. These results are interpretable because we used relevant antigens and followed quality control procedures, including our efforts to validate the PTMs (Appendix A). In addition, these negative findings contrast with our experience with other anti-PTM antibodies that are not routinely seen in clinic, but that we confirmed in patients with RA. These include anti-carbamylated protein antibodies [15,32], anti-acetylated peptide antibodies [16], anti-MAA, and anti-MDA (Rodriguez–Martinez in preparation). Therefore, we conclude that the four atypical anti-PTMs participation are not significant components of RA autoimmunity. This information is noteworthy because it limits the range of recognized autoantigens and sets boundaries for the distinctive features of RA autoimmunity.

We do not know the cause of the disparity between our study and previous ones reporting on the four atypical PTM autoantibodies [9,10,11,12,13,14]. However, the irreproducibility of the unstandardized assays is the prime suspect. The source of irreproducibility could be in the antigens or in the analysis of the ELISA. However, it is also possible that differences between the RA patients played a role. To discuss these issues, we will start by addressing the antecedents, followed by the proteins and peptides used as antigens, the characteristics of the PTMs, the ELISA interpretation, and the patient features.

Previously, three studies have reported the presence of anti-NEG-CII antibodies in RA [11,13,14]. The first study, by Nissim et al. did not include healthy controls but showed a specific reactivity of a magnitude comparable to the one we observed: mean OD = 0.29 vs. 0.20 against NEG-CII and unmodified CII, respectively [11]. The other two studies reported frequencies of anti-NEG-CII antibodies of 64.7% in ERA patients [13], and 32% and 44.4% in established RA patients [13,14]. These frequencies reflect a significant reactivity, whereas Nissim et al. and our low levels of specific anti-NEG-CII antibodies seem inconsequential. Overlapping antecedents apply to the anti-HOCl-CII antibodies because they have been described in two of the same studies [11,13]. The reported frequencies of anti-HOCl-CII antibodies were 92.9% in ERA patients and 32% in established RA patients [13]. The contrast between the absence of specific antibodies in our ERA patients and the reported frequencies is striking. There are two antecedents for the anti-NO_2_ antibodies [10,11]. In the first study, Nissim et al. found low significant reactivity against nitrated CII in patients with RA: mean OD = 0.35 vs. 0.20 against nitrated-CII and unmodified CII, respectively [11]. In the second, Khan et al. reported the presence of antibodies against a nitrated poly-L-tyrosine peptide in all the RA patients analyzed [10]. This study showed some limitations: it was small, and all of the patients, including those with OA and Systemic Lupus Erythematosus (SLE), showed anti-nitrated poly-L-tyrosine antibodies at higher levels than all of the healthy controls. The 100% sensitivity and lack of specificity of the antibodies in this study are remarkable. In turn, the anti-Hcy antibodies have also been reported in two RA studies [9,12]. The first reported anti-Hcy-albumin and anti-Hcy-hemoglobin antibodies [12]. They showed a 21 and 22% increase (mean OD difference = 0.094 and 0.117) for anti-Hcy-albumin and anti-Hcy-hemoglobin, respectively, relative to the healthy controls. The findings of this study, which were uncorrected OD, are comparable to our uncorrected results showing an increase in the OD against Hcy-SynP. The second study reported the prevalence of anti-Hcy-alpha-1-antitrypsin antibodies separately in seronegative RA patients, 75.7%, and seropositive patients, 87.1% [9]. The two subsets of patients differed in their reactivity against native alpha-1-antitrypsin: 1.8% vs. 54.5%, respectively. However, the reactivity against native and modified alpha-1-antitrypsin was analyzed with different protocols, as we will detail below in the ELISA comments. Therefore, some previous reports show results that are comparable to our findings but with less stringent interpretations, whereas others are notably discordant.

The proteins and peptides used as antigens are the first factors that could contribute to the discrepant results. We have used CII from bovine cartilage, the same protein as the previous studies addressing antibodies against the PTMs on CII [11,13,14]. However, CII is a possible source of irreproducibility [33,34]. This large and complex protein suffers changes during purification that could result in the loss of fragments. In addition, it easily carries contaminants, consisting mainly of the molecules that are tightly attached to it [34,35,36]. Some of them, as the proteoglycans, escape detection in the Western blots that are commonly used for purity checks [36]. These properties have led to a wide heterogeneity in the reported frequencies of antibodies against native CII [13,14,33,34,37]. Therefore, we think the variation in CII is a likely factor in our discrepancy with previous results. In addition, there are grounds to suspect the antigens as contributing factors for the differences with the remaining studies. In effect, we used an extract of synovial proteins, SynP, as the base of the nitration and homocysteinylation PTMs. This approach was original. Previously, the anti-NO_2_ antibodies were explored with a NO_2_-CII and a 3-NT-poly-l-tyrosine peptide [10,11], whereas the anti-Hcy antibodies were analyzed using three homocysteinylated serum proteins: albumin, hemoglobin, and alpha-1-antitrypsin [9,12]. However, our preliminary analysis of anti-Hcy-HSA showed low reactivity against this antigen (Supplementary Table S2). In any case, we chose to use SynP, instead, because they are more relevant for RA pathogenesis than serum proteins and more likely to reveal antibodies specific to RA [1,2,3]. In addition, we preferred peptides that are potentially more relevant for RA than the poly-L-tyrosine peptide. Therefore, the choice of different molecules could also be a factor of discrepancy in the anti-nitration and anti-homocysteinylation assays.

Other potential factors are the PTMs themselves because the NEG with ribose, the chlorination with HOCl, and the nitration with peroxynitrite are complex processes that, as detailed in Appendix A, involve multiple reactions [17,18,19,20,21,23]. The three agents induce shared oxidation reactions and other changes specific to each of them. Many modifications are produced in different degrees of abundance, depending on numerous factors. Some of these factors depend on the amino acids upon which they act, including their accessibility and molecular context. As mentioned in Appendix A, we have verified the three PTMs with procedures that included the ones described by Nissim et al. [11]. These analyses showed the presence of the intended PTM and the overall impact of the in vitro reactions. However, the available information for comparison is limited to Nissim et al. [11], is only qualitative, and it only covers a small subset of all the potential changes. Therefore, a certain degree of irreproducibility between the experiments is unavoidable, and it cannot be assessed using the available verification data. In contrast, the nitration in our synthesized peptides is a well-defined 3-NT modification as it was incorporated during synthesis and verified by HPLC and MS. Finally, the in vitro reaction of L-Hcy-thiolactone with proteins produces only N-homocysteinylation [22], a well-defined modification at lysine residues that we verified (Appendix A).

Another putative factor contributing to the discordant results is the analysis of the ELISA because the specific reactivity was determined differently. In the previous studies analyzing anti-modified CII antibodies [11,13,14], the authors subtracted the OD against modified-BSA from the OD against the corresponding modified-CII; i.e., the reported OD of anti-HOCl-CII was the OD obtained against HOCl-CII minus the OD against HOCl-BSA. In our study, we did not correct the results in this way. In its place, we subtracted the OD against unmodified CII, which seems a necessary control in the design of the assay. Accordingly, this correction is the common procedure in the study of the anti-PTM antibodies that is only omitted when the level of reactivity against the unmodified antigens is consistently negligible [4,5,6,7,8,15,32]. The previous studies on modified-CII dealt with the reactivity against the CII backbone by showing that the OD or the frequency of anti-unmodified CII antibodies was lower than that of anti-modified-CII [11,13,14]. It is evident that if the correction for the unmodified-CII had been applied, the previously reported reactivities against the modified CII would have been diminished. In turn, the study by Colasanti et al. compared the reactivity against Hcy-alpha-1-antitrypsin and unmodified alpha-1-antitrypsin [9]. The latter was very notable in the controls and the seropositive RA patients but less marked in the seronegative RA patients. However, this comparison cannot be taken at face value because the ELISA protocols were different. The ELISA for unmodified alpha-1-antitrypsin included two changes that reduce sensitivity relative to the Hcy-alpha-1-antitrypsin ELISA: a higher dilution of serum (1/100 in place of 1/50) and a shorter incubation time of the enzymes’ substrate (5 in place of 20 min). Therefore, an ELISA without these two changes would show very little or no specific reactivity against Hcy-alpha-1-antitrypsin. The other two reports did not analyze unmodified antigens, and they did not correct to establish specificity [10,12]. Therefore, our correction for specificity could be a contributing factor for the discrepant results with all the previous studies.

Finally, our patients might differ from previous patients in the characteristics associated with the atypical antibodies. However, we did not find evidence of this factor. On one side, only two studies identified patient characteristics associated with the antibodies [9,12]. The two studies addressed the anti-Hcy antibodies, but the associated features were different in the two studies and did not replicate in our patients. In one of the studies, the anti-Hcy-albumin or anti-Hcy-hemoglobin antibodies were associated with erosive and active RA but not with the presence of RF or anti-CCP antibodies [12]. On the other, the anti-Hcy-alpha-1-antitrypsin antibodies were more specific for seronegative RA patients [9]. In our study, neither the patients with erosive RA (*p* = 0.1), nor the seronegative RA patients (*p* = 0.7) differ in anti-Hcy-SynP reactivity. On another side, we studied anti-NEG-CII and anti-HOCl-CII antibodies in ERA patients, the group with the reported higher frequencies [13]. As a piece of further evidence, we found that our ERA patients did not show any significant correlation between the anti-HOCl-CII or anti-NEG-CII antibody levels and disease activity as assessed with the DAS28, or the IgG total levels; they also showed similar anti-HOCl-CII and anti-NEG-CII antibody levels in all of the strata made by comparing treatment with the absence of treatment with GC, NSAID, MTX, other csDMARD, bDMARD or anyDMARD. Despite the lack of evidence on the patients’ role, it is worth commenting on the lower frequency of smokers among our patients in comparison with the patients in Xie et al. (30% vs. 62% [14]; frequency not reported by other studies) because smoking predisposes to autoantibody production in RA [38,39]. Finally, although there was little information on the patient characteristics in the nitrated poly-L-tyrosine study [10], no particular feature is expected because 100% of the RA, OA, and SLE patients were antibody positive.

Our study has tried to replicate previous findings to gain knowledge on RA. Therefore, we chose relevant antigens and used reliable protocols to produce and verify the PTM and to run and interpret the ELISA. For example, we assessed the glycation and chlorination PTM following the protocols described by Nissim et al. [11], which were also followed by Strollo et al. and Xie et al. [13,14]. In addition, we used the same commercial source of high purity CII as Xie et al. [14] and we included the verification steps described by Nissim et al. [11]. However, we changed the correction for specificity because it was necessary according to the experience with the best established RA anti-PTM antibodies [4,5,6,7,8,15,32]. Therefore, we propose that the four atypical antibodies are either unspecific of the PTM or present at low levels of uncertain meaning. We hope other researchers will test the reactivity against these four PTMs to confirm our results.

## 5. Conclusions

Our study has found that the antibodies targeting four PTMs: chlorination with HOCl, NEG with ribose, nitration with peroxynitrite, and homocysteinylation with L-Hcy-thiolactone, are absent or inconsequential in patients with RA. This outcome implies that the range of PTMs able to trigger autoimmunity in RA patients is restricted and calls for experiments to understand their defining features.

## Figures and Tables

**Table 1:** Clinical characteristics of the two subsets of patients included in the present study ^1^.

	Early RA ^2^ *n* = 182	Established RA ^2^ *n* = 143
Age, median (IQR) ^3^ years	58 (48–71)	61 (50–72)
Age of diagnosis, median (IQR) years	58 (48–71)	47 (35–56)
Symptom duration, median (IQR) years	0 (0–1)	11 (5–21)
Women, n (%)	112 (62)	112 (78)
Ever smoked, n (%)	54 (30)	23 (18)
Erosions, n (%)	17 (14)	92 (66)
RF positive, n (%)	94 (56)	84 (61)
Anti-CCP positive, n (%)	119 (65)	97 (68)
Anti-CarP positive, n (%)	63 (41)	48 (34)
DMARD naïve, n (%)	109 (82)	-
MTX, n (%)	6/133 (5)	-
Other csDMARD, n (%)	10/129 (8)	-
bDMARD, n (%)	8/133 (6)	-
Glucocorticoids, n (%)	115/122 (94)	-
NSAIDs, n (%)	83/113 (73)	-
DAS28, median (IQR)	4.94 (3.53–5.97)	-
IgG [mg/dL], median (IQR)	1195 (1010–1440)	-

^1^ The healthy controls were 60% women with a median age = 69 years, IQR = 63–75. ^2^ Information was available for <95% early RA patients: erosions = 69%; RF = 92%; and anti-CarP = 85%; treatments = 62–73%; DAS28 = 68%; total IgG (mg/dL) = 65%. For <95% established RA patients: smoking = 91%. ^3^ Abbreviations: IQR = interquartile range; RF = rheumatoid factor; CCP = Cyclic Citrullinated Peptide; CarP = Carbamylated Proteins; DMARD = Disease Modifying Anti-Rheumatic Drug; MTX = methotrexate; other csDMARD = conventional synthetic DMARD different from MTX; bDMARD = biologic DMARD; DAS28 = Disease Activity Score 28 joints.

**Table 2:** Summary of the four analyzed PTMs.

PTM	Protein	Modifying Agent	Abbreviation
chlorination	collagen type II	hipochlorous acid	HOCl-CII
glycation	collagen type II	ribose	NEG-CII
nitration	synovial proteins	sodium peroxinitrite	NO_2_-SynP
nitration	8 peptides	synthesis	3-NT-pep
homocysteinylation	synovial proteins	L-homocysteine-thiolactone	Hcy-SynP

**Table 3:** The 10 synthetized 3-NT peptides with an indication of their position in the protein sequences, the nitrated residues (3-NO_2_-Y), and the probabilities of in vivo nitration according to two algorithms.

Protein	Sequence	Δ ^1^	Score ^2^
Enolase 1_247–267_	AASEFFRSGK(3-NO_2_-Y)DLDFKSPDDP	0.999	2.881
Enolase 2_34–54_	AVPSGASTGI(3-NO_2_-Y)EALELRDNDK	0.584	1.498
Fibrinogen_312–332_	GKNYCGLPGE(3-NO_2_-Y)WLGNDKISQL	0.903	1.611
Vimentin_20–40_	TASRPSSSRS(3-NO_2_-Y)VTTSTRTYSL	0.963	2.693
14-3-3 η_206–226_	AELDTLNEDS(3-NO_2_-Y)KDSTLIMQLL	1.061	1.695
BiP_165–185_ ^3^	VTHAVVTVPA(3-NO_2_-Y)FNDAQRQATK	3.237	2.824
Tenascin_1676–1696_ ^3^	DITGLREATE(3-NO_2_-Y)EIELYGISKG	0.457	0.912
Histone H3_32–52_	ATGGVKKPHR(3-NO_2_-Y)RPGTVALREI	1.319	1.679
Histone H4 1_63–83_	LENVIRDAVT(3-NO_2_-Y)TEHAKRKTVT	0.731	1.866
Histone H4 2_42–62_	GGVKRISGLI(3-NO_2_-Y)YEETRGVLKVF	0.546	1.448

^1^ iNitro-Tyr prediction. ^2^ GPS-YNO2 1.0 prediction. ^3^ Discarded peptides due to low purity.

**Figure A1:**
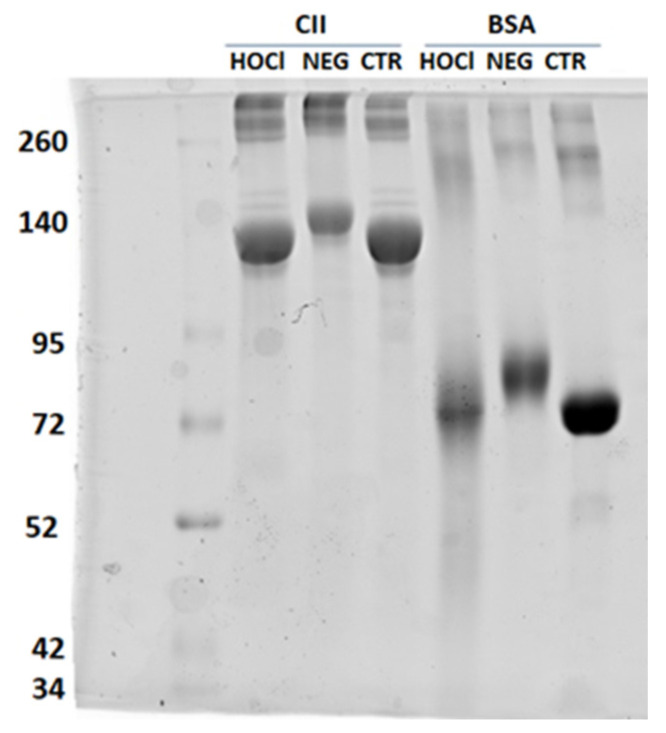
SDS-PAGE analysis of the HOCl-CII and NEG-CII compared with the unmodified CII and the control changes in BSA. The numbers on the left indicate the marker MW (kDa).

**Figure A2:**
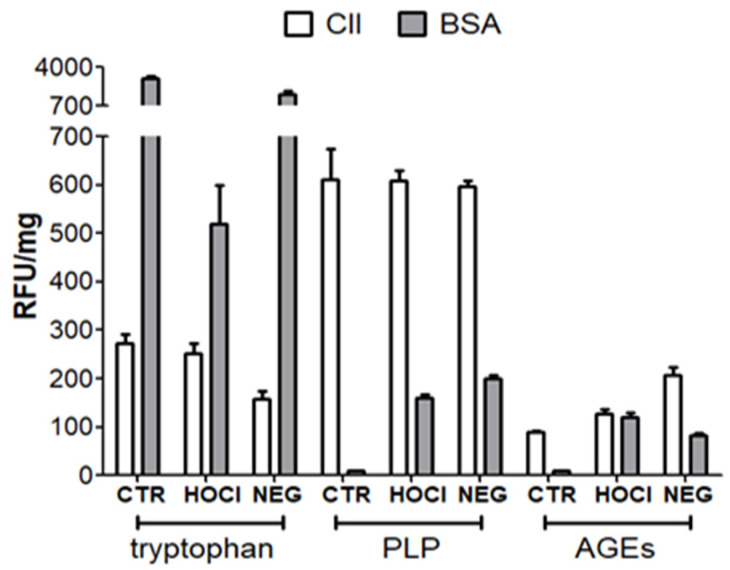
Fluorescence intensity of HOCl-CII and NEG-CII at wavelengths to detect tryptophan, PLP and AGEs compared with unmodified CII and the control changes in BSA. The bars represent the mean and standard error of the mean (SEM) of at least 3 independent experiments.

**Figure A3:**
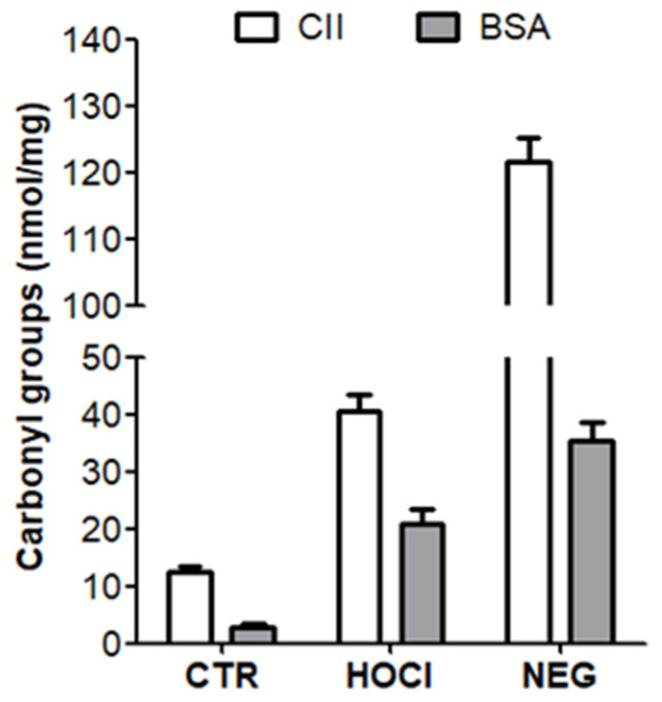
Carbonyl concentration in HOCl-CII and NEG-CII compared with the unmodified CII and the control changes in BSA. The bars represent the mean and SEM of at least 4 independent experiments.

**Figure A4:**
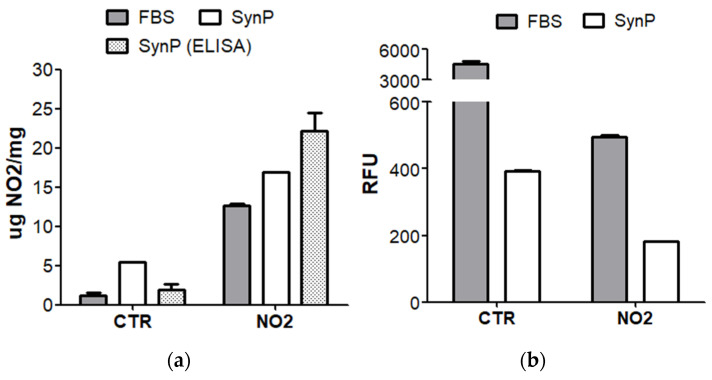
Assessment of NO_2_-SynP in comparison with unmodified SynP and control FBS by (**a**) absorbance and ELISA to detect 3-NT; and (**b**) fluorescence to detect unaltered tyrosine. Th bars represent the mean and SEM of at least 2 independent experiments except for the absorbance measurement that was performed once.

## Data Availability

The data presented in this study are available on request from the corresponding author.

## Supplementary Materials

The following supporting information can be downloaded at: https://www.mdpi.com/article/10.3390/diagnostics12020352/s1, Table S1: OD obtained in the ELISA for the four anti-PTM antibodies in the patients with RA and the controls. Table S2: OD obtained in the preliminary ELISA for the Hcy PTM using anti-Hcy-HSA antigen.

## Appendix A

### Appendix A.1. Nature of the PTM Induced by HOCl, Ribose and Peroxynitrite

The production of the three PTMs: chlorination with HOCl, NEG with ribose, and nitration with peroxynitrite, has several aspects in common [17,18,19,20,38,39]. The incubation of the proteins with the three reagents, (1) induces complex reactions that modify a variety of amino acid residues and lead to a range of derivatives; (2) a shared component of oxidation reactions; (3) many derivatives are reactive intermediates that induce new protein modifications; (4) the final effect includes protein structural changes; and (5) the three PTMs are present at increased levels in the RA patients [9,17,22,26].

Here, we describe the shared component of oxidation reactions and, in the following paragraphs, the differential component of reactions in each of the three PTMs [17,18,19,20,38,39]. The amino acids that are particularly susceptible to oxidation are cysteine, methionine, tryptophan, and tyrosine. Oxidation of the cysteine thiol leads to many modifications, depending on their accessibility in the protein and the oxidant: cysteine sulfenic, sulfinic, and sulfonic acid derivatives, in addition to the formation of inter- or intra-molecular disulfide bonds with another free thiol. Further, the series of oxidation derivatives of tryptophan is notable. In turn, several oxidants induce the tyrosyl radical that easily forms stable inter- or intra-molecular cross-links through di-tyrosine with another tyrosyl radical. Another common modification that arises via multiple mechanisms is protein carbonylation. This modification can result from a direct oxidative attack on proline, arginine, lysine, and threonine. Alternatively, carbonyls arise via the secondary reaction of lysine, cysteine, and histidine with reactive carbonyls, including those derived from NEG. Finally, structural damage to the proteins can lead to their fragmentation and the formation of aggregates. The aggregates result from the already mentioned disulfide bridges, tyrosine cross-links, and other mechanisms. The shared changes vary in abundance depending on the modifying agent and other parameters.

In the case of chlorination, HOCl produces a wide array of the shared oxidation reactions plus some specific derivatives. For example, HOCl modifies cysteine to sulfenyl chloride, a distinctive modification, in addition to the shared ones: sulfenic, sulfinic, and sulfonic acids, and disulfide. In addition, HOCl reacts with tyrosine to yield 3-chloro-tyrosine and 3,5-dichloro-tyrosine. Another specific modification is the formation of chloramines on lysine, histidine, and arginine, and chloramides on glutamine and asparagine, respectively. Chloramines and chloramides are reactive intermediates that extend protein modifications.

Similarly, glycation induces a wide range of molecular changes, many of them glycoxidations. The NEG with ribose (or other sugars) gives rise to a highly heterogeneous class of stable adducts, the AGEs. Glycation is a three-stage process: (1) the reversible condensation of the sugar aldehyde with a protein amino group to form a Schiff base; (2) the Schiff base rearrangement and condensation to yield an Amadori intermediate; (3) the intermediate cycles of condensation to produce AGEs. The Schiff bases and some Amadori intermediates participate in glycoxidation. In addition, one of the outcomes of glycoxidation is protein carbonylation, as when the α-carbonyls (Amadori intermediates) modify the basic residues of lysine and arginine. Further, two of the predominant AGEs, carboxymethyl lysine (CML) and pentosidine, are formed via glycoxidation.

In addition, the treatment of proteins with peroxynitrite induces an array of modifications that include the shared oxidations and the specific nitration of tyrosine residues. Some of the oxidation reactions are abundant as are the oxidation of cysteine, tryptophan, and methionine and the production of carbonyls. The nitration of tyrosine readily yields 3-nitrotyrosine (3-NT), which has become a biomarker of the effect of peroxynitrite. Tyrosine nitration can also produce 3,3′-di-tyrosine that favors the cross-linked protein aggregates. Another remarkable characteristic of protein tyrosine nitration is that it occurs only in a few tyrosine residues, typically one or two, per protein, and not in all the proteins.

### Appendix A.2. Verification of the PTM Induced by HOCl and Ribose

We used three techniques to verify the changes induced in HOCl-CII and NEG-CII: sodium-dodecyl sulfate-polyacrylamide gel electrophoresis (SDS-PAGE), fluorescence analysis, and quantification of the carbonyls (Table A1).

**Table A1:** Techniques used for verification of the four PTMs.

Antigen	Assay	Information
HOCl-CII & NEG-CII	SDS-PAGE	Integrity, apparent MW, aggregates
	Fluorescence	Tryptophan, PLP, other AGE
	DNPH	Carbonyls
NO_2_-SynP	Absorbance	3-NT (low specificity)
	ELISA	3-NT (high specificity)
	Fluorescence	Tyrosine (unmodified)
Hcy-SynP	Competitive ELISA	Total homocysteine
3-NT-pep ^1^	HPLC	Purity, elution time
	MS	Purity and peptide mass

^1^ These procedures were performed by the peptide provider and are not described here.

We assessed the integrity, apparent molecular weight (MW), and presence of aggregates using SDS-PAGE (Figure A1). The unmodified CII showed a principal band at ≈130 kDa and some bands of higher MW. The ≈130 kDa band corresponds to the α-chain of bovine CII, which has an apparent MW greater than its real MW (95 kDa) due to its fibrillar structure. Chlorination with HOCl produced only minor changes: some large aggregates that remained in the loading well and a mild increase in the intensity of the ≈65 kDa bands that could represent CII fragmentation. Nissim et al. reported the same changes [11]. However, the HOCl-BSA used as the control showed more marked alterations. In turn, the NEG modification of CII with ribose leads to a shift in all of the bands towards a higher apparent MW. This change in mobility is due to the glycation process. There was also a general decrease in the intensity of the <200 kDa bands and increase in the >200 kDa bands that could represent the formation of aggregates. The control NEG-BSA showed a similar shift and band intensity changes. Nissim et al. also reported a NEG-CII shift to a higher MW, but these authors found new CII fragmentation bands that were absent in our preparation [11].

The second technique consisted of the fluorescence 2D analysis after excitation at three wavelengths (Figure A2) [40,41,42]. The three excitation wavelengths were: 295 nm to estimate tryptophan with a small contribution from tyrosine and phenylalanine; 335 nm for pentosidine-like products (PLP); and 370 nm for other AGEs. We recorded the emission spectra using an F-2500 FL spectrofluorometer (Hitachi, UK) in the range of 230–600 nm. The maximum fluorescence intensity was taken as relative fluorescence units and corrected for the protein mass (RFU/mg).

These analyses showed a significant level of PLP already present in the unmodified CII that did not increase with chlorination or NEG (Figure A2). However, PLP were notably increased in BSA after the two transformations. In addition, the chlorination with HOCl almost did not decrease the level of tryptophan in CII, but increased 1.4 times the level of fluorescence in the wavelength of AGEs (maximum emission at 425 nm). These mild changes do not indicate ineffective HOCl, because it produced marked alterations in BSA. In addition, they are similar to the almost absence of changes reported by Nissim et al. [11]. The NEG with ribose, in contrast, markedly decreased the tryptophan signal to about half the initial level (Figure A2). This decrease is similar to that observed on the BSA. In turn, the NEG also increased 2.3 times the fluorescence of AGE at 425 nm. The fluorescence of AGE on the BSA reaction was also increased but with an emission maximum at 440 nm indicating a different set of adducts.

Finally, the carbonyl content of HOCl-CII and NEG-CII was quantified using the 2,4-dinitrophenylhydrazine (DNPH) assay (Figure A3) [43,44]. Briefly, 250 µg of the antigens were precipitated in 10% trichloroacetic acid (TCA), dissolved in 50 µL of 5% SDS and incubated with 200 µL of 10 mM DNPH in 2.5 M HCl and 0.5 M H3PO4 for 1 h in the dark with stirring. The reaction was terminated by TCA precipitation, washed with ethanol-ethyl acetate to remove free DNPH, and dissolved in 6 M guanidine-HCl pH 2.5. Light absorbance of the supernatant was measured at 370 nm and quantified using the molar extinction coefficient of the protein-hydrazone complex (22.000 M^−1^cm^−1^). The carbonyl content increased from 12.6 nmol/mg protein in the unmodified CII to 40.5 nmol/mg in HOCl-CII and 121.6 nmol/mg in NEG-CII. Similarly, the carbonyl content of BSA increased from 3.0 to 21.1 and 35.6 nmol/mg, respectively.

### Appendix A.3. Verification of the Nitration of Proteins with Peroxynitrite

For verification of the NO_2_-SynP, we used three techniques (Table A1). First, we used light absorbance at 420 nm, the absorbance maximum of 3-NT at pH 9.0, as unmodified tyrosine does not absorb in these conditions [10]. We used a calibration curve made with commercial 3-NT (Merck, Germany). We tested four concentrations of peroxynitrite on FBS in our preliminary experiments. The maximum formation of 3-NT, 13 µg 3-NT/mg FBS, was obtained with 10 mM peroxynitrite, when the basal level was 1 µg 3-NT/mg in the unmodified FBS. We used this concentration to produce NO_2_-SynP, which showed an increase from 5.5 µg 3-NT/mg in the unmodified SynP to 17.0 µg 3-NT/mg in NO_2_-SynP (Figure A4a). As we suspected, the absorbance obtained with the unmodified SynP could be due to other substances in the protein extract; we used a more specific test. It consisted of quantification with an ELISA kit that includes an antibody specific for 3-NT (#ab116691, Abcam, UK). This determination showed a larger difference between NO_2_-SynP and unmodified SynP, 22.0 vs. 1.9 µg 3-NT/mg protein, respectively (Figure A4). Finally, we used fluorescence to assess the tyrosines that remained after nitration because 3-NT does not emit fluorescence in the assay conditions [45]. We performed the analysis at pH 9 on a quartz cuvette excited at 275 nm, recording the maximum emission in the 360–450 nm range. NO_2_-SynP showed a significant decrease in fluorescence, but there was still some emission over the level of the blank, suggesting that there were unmodified tyrosines or other fluorescent substances (Figure A4b). The FBS showed a notably higher fluorescence level in the unmodified status that was also significantly decreased by nitration.

### Appendix A.4. Nature and Verification of Homocysteinylation

The interest in the homocysteinylation of proteins comes from cardiovascular diseases, where high homocysteinemia has been considered a risk factor [21]. The two reports of antibodies to anti-homocysteinylated proteins in RA used protein antigens that were modified by a reaction with L-Hcy-thiolactone [9,12]. We followed the same procedure. This precision is necessary because other homocysteinylation reactions lead to differentially modified proteins [21]. The L-Hcy-thiolactone reaction is a non-enzymatic process that involves the acylation of the ε-amino group of lysine residues. However, this modification is restricted to a few lysine residues in each protein for unknown reasons. This selectivity likely makes the anti-Hcy reactivity sensitive to the protocol and protein used.

We evaluated the amount of Hcy incorporated into SynP with a clinically validated competitive immunoassay that quantifies total Hcy (IMMULITE^®^ 2000 Homocysteine, Siemens). This assay uses a monoclonal antibody specific for S-adenosyl-L-Hcy (SAH), an enzymatically reduced form of Hcy. We tested four reaction conditions combining concentrations of L-Hcy-thiolactone and times of incubation. The maximum level of Hcy incorporation without protein damage was obtained at 1 mM for 16 h leading to 2.26 nmol Hcy/mg of protein. The unmodified SynP showed no detectable Hcy. We used these preparations in our ELISA.
