# Supplementary material for: Antibodies against 4 Atypical Post-Translational Protein Modifications in Patients with Rheumatoid Arthritis
Source: Diagnostics (Basel). 2022 Jan 29;12(2):352. doi: 10.3390/diagnostics12020352 (PMC8870974; doi:10.3390/diagnostics12020352)
Supplement: Supplementary file 1 [file diagnostics-12-00352-s001.zip › diagnostics-1531540-supplementary.pdf]

Supplementary Table S1

OD obtained in the ELISA for the four anti-PTM antibodies in the patients with RA and the controls. The results corresponding to each antigen with and without correction are shown as median values and the interquartile range (IQR). The comparison was done with the U of Mann-Whitney test.

		Controls		RA patients		
Antigen[1]	Correction[2]	Median	IQR	Median	IQR	*P*
ctr-CII	-	0.174	0.147/0.201	0.143	0.128/0.169	$5.0x10^{-9}$
HOCl-CII	no	0.160	0.142/0.187	0.141	0.127/0.172	$3.1x10^{-5}$
HOCl-CII	yes	-0.004	-0.020/0.005	-0.002	-0.011/0.008	0.01
NEG-CII	no	0.221	0.163/0.305	0.234	0.171/0.285	0.5
NEG-CII	yes	0.027	-0.010/0.099	0.072	0.016/0.119	0.0003
ctr-SynP	-	0.020	0.008/0.026	0.022	0.009/0.033	0.1
NO2-SynP	no	0.011	0.008/0.035	0.018	0.009/0.028	0.1
NO2-SynP	yes	-0.011	-0.031/0.009	-0.009	-0.022/0.005	0.7
ctr-pep	-	0.097	0.090/0.106	0.111	0.089/0.150	0.02
3-NT-pep	no	0.209	0.190/0.230	0.250	0.201/0.329	0.02
3-NT-pep	yes	0.113	0.100/0.133	0.137	0.097/0.185	0.1
ctr-SynP	-	0.186	0.171/0.221	0.211	0.181/0.229	0.04
Hcy-SynP	no	0.145	0.130/0.155	0.160	0.145/0.178	0.0001
Hcy-SynP	yes	-0.038	-0.069/-0.020	-0.041	-0.074/-0.015	0.5

[1] Abbreviations: ctr = unmodified control protein or peptide; CII = collagen type II; HOCl = chlorination with hypochlorous acid; NEG = non-enzymatic glycation with ribose; SynP = synovial proteins; NO2 = nitration with peroxynitrite; pep = peptides; 3-NT = 3-nitrotyrosine; Hcy = homocysteinylation with homocysteine-thiolactone.

[2] Correction consisted in subtracting the OD against the unmodified protein or peptide from the OD against the corresponding modified protein or peptide for each subject.

Supplementary Table S2

OD obtained in the preliminary ELISA for anti-Hcy-HSA antibodies. The samples correspond to 38 patients with established RA and 37 healthy controls. The results are shown as median values and the interquartile range (IQR). The comparison was done with the U of Mann-Whitney test.

		Controls		RA patients		
Antigen[1]	Correction[2]	Median	IQR	Median	IQR	*P*
ctr-HSA	-	0.179	0.139/0.262	0.182	0.152/0.281	0.8
Hcy-HSA[3]	no	0.171	0.147/0.250	0.186	0.146/0.264	0.6
Hcy-HSA	yes	-0.006	-0.018/0.007	0.001	-0.003/0.011	0.02

[1] Abbreviations: ctr = unmodified control protein or peptide; HSA = human serum albumin; Hcy = homocysteinylation with homocysteine-thiolactone.
[2] Correction consisted in subtracting the OD against the unmodified protein from the OD against the corresponding modified protein for each subject.
[3] HSA (Sigma-Merck, A8763) was reacted with 0.4 mM L-Hcy-thiolactone as described (Nowakowska-Plaza *et al*. 2014) leading to 0.81 nmol Hcy/mg protein without protein damage.
